# Basic counseling skills in psychology and teaching: validation of a short version of the counselor activity self-efficacy scales

**DOI:** 10.1186/s40359-023-01506-7

**Published:** 2024-01-18

**Authors:** Joanna Joy Hunsmann, Destina Sevde Ay-Bryson, Scarlett Kobs, Nicole Behrend, Florian Weck, Michel Knigge, Franziska Kühne

**Affiliations:** 1 Department of Psychology, Clinical Psychology and Psychotherapy, University of Potsdam, Potsdam, Germany; 2 Department of Rehabilitation Sciences, Rehabilitation Psychology, Humboldt-Universität, Berlin, Germany; 3 Department of Inclusive Education, Inclusive School and Instructional Development, University of Potsdam, Potsdam, Germany; 4 Department of Psychology, Counseling Psychology, University of Potsdam, Potsdam, Germany

**Keywords:** Counselor self-efficacy, Skills, Training, Communication, Supervision

## Abstract

**Background:**

Counseling self-efficacy is a relevant measure to examine trainees’ beliefs about their counseling skills. This study aimed to validate three scales of the revised German version of the Counselor Activity Self-Efficacy Scales (CASES-R) measuring basic counseling skills. To ascertain the scales’ sensitivity to change, counseling self-efficacy was assessed before and after specific training.

**Method:**

The sample comprised 163 university students enrolled either in psychology or education. Students were examined before and after participating in training focusing on basic counseling skills. We applied confirmatory factor analysis and tested internal consistency, convergent validity, and criterion validity.

**Results:**

Confirmatory factor analysis supported the three-factor structure of the CASES-R scales for basic counseling skills. The scales provided acceptable to good internal consistency (α = 0.77 − 0.87). Significant relations with general self-efficacy (*r* =.23, *p* <.01) provided first indication for convergent validity. We also found a significant correlation of the CASES-R with positive affect (*r* =.22), and significant correlations of some subscales with empathetic concern (*r* =.16 −.21) and mastery goal orientation (*r* =.16), overall supporting criterion validity. The CASES-R scales proved to be sensitive to change, as participants’ scores were higher after (*M* = 6.18, *SD* = 1.05) than before (*M* = 5.37, *SD* = 1.16) counseling training (*F*(1, 309) = 42.27, *p* <.001).

**Conclusion:**

We found support for the proposed factor structure and reliability of the German version of the three CASES-R scales, indicating its suitability for use in basic counseling settings. Future research should further examine the scales’ validity.

**Supplementary Information:**

The online version contains supplementary material available at 10.1186/s40359-023-01506-7.

Counseling self-efficacy (CSE) can be defined as a counselor’s “beliefs or judgements about his or her capabilities to effectively counsel a client in the near future” [1]. According to Bandura [2], individuals with higher self-efficacy are generally more willing to approach difficult tasks, expend more energy, show more endurance when encountering challenges, and remain confident despite failures. Self-efficacy in literature has been widely linked to better performance [3, 4]. General self-efficacy (GSE), a global measure of self-efficacy, has been shown to predict domain-specific self-efficacies such as CSE [4] and occupational self-efficacy [5], and is considered especially influential when individuals engage in unfamiliar tasks [6].

As a domain-specific self-efficacy, the development of CSE is important for prospective counselors as they learn new counseling skills and aspire to successfully perform counseling-related tasks [7]. CSE has been related to more positive and lower negative affectivity toward counseling-related activities [8], empathetic concern and mindfulness [9], and goal orientation in counseling [10]. Furthermore, CSE has been inversely related to anxiety [1] and stress [9] in counselors. In studies with professional counselors, CSE has been linked to better performance and higher competence, such as the skillful use of emotions during problem solving in counseling sessions [11] and more active engagement with difficult counseling situations [10, 12]. Further benefits of CSE include higher job satisfaction and lower levels of risk for counselor burnout [13, 14].

Investigating its growth in counseling students, CSE has been found to increase with training in master’s- and doctoral-level counseling students in the United States [15] and be positively impacted by practice and clinical supervision [16, 17]. However, in a longitudinal study on CSE development in master’s level students enrolled in a counseling program in the United States, Mullen and colleagues [18] found that CSE increased most markedly in early counseling education, even before students entered clinical training and practical internships. Goreczny and colleagues [17], on the other hand, suggest a curvilinear development in early stages of training: Their findings from a university in the United States indicate that CSE may initially be quite high among psychology students, only to sink low as first practical experiences are made before they eventually increase over time and under the influence of practice and supervision [17]. While these findings highlight the importance of early training stages for the formation of CSE, they also demonstrate that more research is needed, calling for the development of a measure to specifically assess CSE at beginner levels of counseling training.

As one such measure, the Counselor Activity Self-Efficacy Scales (CASES) were developed in the United States by Lent and colleagues [10]. Conceptually based on the Helping Skills Model [19], existing research, and their professional experience, the authors aimed to precisely assess CSE across a broad range of skill levels, addressing CSE in managing routine counseling tasks as well as in dealing with advanced counseling situations [10]. The CASES contain three conceptually derived subdomains: *Helping Skill Self-Efficacy* (HS), *Session Management Self-Efficacy* (SM), and *Counseling Challenges Self-Efficacy* (CC, see Fig. 1).

**Fig. 1:**
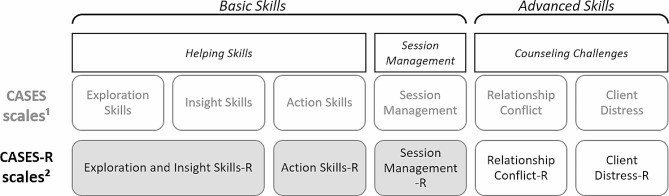
Overview of the subdomains and scales of the CASES and the revised CASES-R. ¹Lent et al. [10]; ²Hahn et al. [4]. Scales applied in the current study are displayed in gray

Items generated by the authors, based on these aspects, were reviewed by several practicing professionals, resulting in an initial version of the instrument with 59 items. Exploratory factor analyses were conducted separately for each subdomain, yielding the factor structure of the CASES. The HS subdomain items yielded the scales *Exploration Skills* (ES; e.g., reflecting feelings), *Insight Skills* (IS; e.g., challenging client inconsistencies), and *Action Skills* (AS; e.g., homework assignments). Items from the subdomain SM loaded on one factor, resulting in the scale *Session Management* (SM; e.g., keeping track of time). The CC subdomain yielded the two scales *Relationship Conflict* (RC; e.g., solving interpersonal tensions between client and counselor) and *Client Distress* (CD; e.g., working with a clinically depressed client). Second-order analyses of the six scales supported the conceptual distinction between self-efficacy in basic counseling skills, containing the *Insight Skills*, *Exploration Skills*, *Action Skills*, and *Session Management* scales, and advanced counseling skills, reflected by the *Client Distress* and *Relationship Conflict* scales. Cronbach’s α coefficients indicated acceptable to excellent internal consistency of the scales (0.79 ≤ α ≥ 0.97) with an adequate two-week test-retest reliability (*r* =.59 −.75). Convergent and discriminant validity were confirmed using existing measures of CSE and social desirability, respectively. Negative correlations with negative affect and positive correlations with positive affect towards the role of the counselor provided evidence for criterion validity. The CASES were shown to be sensitive to change, as students’ CSE increased over the course of their studies, during which they received counseling training and gained practical experience [15].

Recognizing the need for more culture-specific research on CSE, Hahn and colleagues [4] translated the CASES into German and validated the translated instrument. Given that the three subdomains of the CASES, namely HS, SM, and CC, were derived by Lent and colleagues [10] conceptually but not confirmed statistically, they reexamined the factor structure in detail. Confirmatory factor analysis of the translation with a German sample of postgraduate psychotherapists in training did not yield satisfactory results, leading the authors to conduct further exploratory factor analyses (EFA) and structural equation modelling (ESEM) with a new sample. After stepwise elimination of problematic items, i.e., due to substantial cross-loadings, the authors found support for a five-factor solution, the best fit being a bifactor-ESEM model with one general and five specific factors. The translated and thus shortened revised CASES-R therefore contains five subscales, labeled as *Exploration and Insight Skills-Revised* (EIS-R), *Action Skills-Revised* (AS-R), *Session Management-Revised* (SM-R), *Relationship Conflict-Revised* (RC-R), and *Client Distress-Revised* (CD-R).

To improve counseling training, more research specifically targeting and monitoring CSE in novice counselors and educators is needed. Lent and colleagues [10] specified that the CASES scales can be used separately, and researchers demonstrated this in a study on ethnic diversity in the classroom with a mixed sample of 402 students enrolled in either psychology or education [20] or in a study with 110 prospective counselors [15]. Here, higher CSE in *Helping Skills* and *Session Management* was associated with greater congruence between client and counselor ratings of session quality [15].

Recognizing the importance of accurately assessing and monitoring CSE in early training stages, the current research aimed to validate those CASES-R scales that target trainees’ perceived ability in basic counseling skills. Thus, we aimed to examine the psychometric properties of the EIS-R, AS-R, and SM-R subscales, using a sample of German undergraduate students of education and master’s level students of psychology undergoing helping skills courses. As the CD-R and RC-R scales of the CASES-R reflect the engagement with advanced counseling situations, they were not included in this research with trainees at a beginner level.

The first objective of the current study was to examine the factor structure of the first part of the CASES-R, which relates to self-efficacy of basic counseling skills. In line with Hahn et al. [4], we expected three factors to emerge: Exploration and Insight (EIS-R), Action Skills (AS-R), and Session Management (SM-R). The second objective was to investigate its reliability, whereby we hypothesized at least acceptable internal consistencies. Third, we examined the instrument’s validity, and expected significant positive correlations with general self-efficacy (convergent validity), significant positive correlations with positive affect, empathic concern, and motivation to improve one’s counseling skills (criterion validity), and finally significant negative correlations with negative affect (criterion validity). Our fourth aim was to explore the scales’ sensitivity to change, i.e., counseling self-efficacy at the beginning and at the end of specific training, whereas we expected higher values after training.

## Methods

### Sample

The sample comprised undergraduate students who were enrolled in special education or rehabilitation education at the Humboldt-University of Berlin (HU) and master’s level psychology students who were enrolled at the University of Potsdam (UP). Each education student participated in one of five courses on counseling practice (HU: *n* = 15, *n* = 23, *n* = 20, *n* = 20, *n* = 28, each) and each psychology student participated in one of four courses to acquire skills in psychological counseling (UP: *n* = 17, *n* = 14, *n* = 15, *n* = 11, each). The nine courses were part of the students’ regular curriculum and were held during the summer semester in 2022, which in Germany begins in April and ends in July. We invited all students enrolled in the courses to participate in our study and thus recruited a convenience sample. Some of the courses took place weekly throughout the university term, and others as block events at the end of the term.

### Training

As for the content, each course encompassed theoretical introductions on basic counseling skills and practical exercises to apply the newly learned techniques. The training was informed by core counseling skills present in the CASES-R. The theoretical content of the courses entailed providing a framework of communication skills, the introduction of specific techniques such as paraphrasing (reflecting the third item of the CASES-R), reflecting emotions (referring to the fifth item), or validation, as well as guidance for structuring and managing counseling sessions, e.g., keeping them on track and focused. Further, during the classes nonverbal communication was introduced as part of professional counseling techniques, which included the role of a counselor’s attending, reflecting the first item of the CASES-R. Another example is that the theoretical input offered students knowledge and insight regarding possible biases. This enabled students to be mindful of interpreting what clients say, reflecting item six of the CASES-R. Practical exercises following theoretical input were conducted as role-plays between students and provided opportunities to regularly receive specific feedback from peers and lecturers. Role-play in the counseling setting was yet another skill reflected in the CASES-R (referring to item eight). Performance in class was not graded.

### Procedure

Participation in the study was voluntary, there was no reimbursement, and all participants gave informed consent. Students who were aged 18 and above were eligible to participate in the study. Participants completed the questionnaire containing a small number of sociodemographic questions, as well as a fixed set of measures described below, during the first or second session of their course and again upon completion of their course. A total of *N* = 163 students (*n* = 135, 82.8% female; *n* = 27, 16.6% male; *n* = 1, 0.6% not specified) completed the questionnaire at the beginning of the semester, and *N* = 157 (*n* = 128, 81.5% female; *n* = 25, 15.9% male; *n* = 1, 0.6% diverse; *n* = 3, 1.9% not specified) provided data three months later. At the first measurement, participants indicated their age as follows: 18–20 years old: *n* = 16, 9.8%, 21–30 years old: *n* = 116, 71.2%, 31–40 years old: *n* = 15, 9.2%, 41–50 years old: *n* = 4, 2.5%, not specified: *n* = 12, 7.4%.

### Measures

#### Counselor activity self-efficacy scales-revised (CASES-R)

As the focus of the current study was to assess counseling skills at beginner level, we used three of the five CASES-R scales [4], which apply to CSE in basic counseling skills, that is *Exploration and Insight Skills-Revised* (EIS-R; to explore the client’s understanding of a problem, e.g., *restatements*: to paraphrase the words of a client in a specific and concise manner), *Action Skills-Revised* (AS-R; the ability to apply a structured intervention, e.g., *direct guidance*: suggest, direct, or advise a client towards specific actions), and *Session Management-Revised* (SM-R; the ability to manage counseling sessions, e.g., by *keeping sessions on track*: maintain direction and concentration). For an overview of the CASES-R items, see Supplementary Material 1. The items are rated on a 10-point Likert scale from 0 (*no confidence at all*) to 9 (*complete confidence*), with higher scores indicating higher CSE [10]. The internal consistency of the CASES-R has been reported to be overall good, with an omega hierarchical coefficient of *ωH* = 0.79, while individual subscales had lower internal consistencies (EIS-R: *ωH* = 0.36, AS-R: *ωH* = 0.64, SM-R: *ωH* = 0.31) [4]. The omega hierarchical coefficient considers the hierarchical structure of variables and accounts for both common and unique factors.

#### Convergent validity

To examine convergent validity, we used the German *General Self-Efficacy Scale* [21]. The GSE assesses the optimistic self-belief to cope with various challenging situations (e.g., “I am confident that I could deal efficiently with unexpected events”). The scale comprises ten items on a 4-point Likert scale ranging from 1 (*not at all true*) to 4 (*exactly true*), with higher values indicating higher GSE. In the current sample, Cronbach’s α was 0.74.

#### Criterion validity

We applied German version [22] of the *Interpersonal Reactivity Index* (IRI) [23] to measure participants’ empathic concern, that is a feeling of compassion for someone in an adverse situation (e.g.: “Before criticizing somebody, I try to imagine how I would feel if I were in their place.”). Its 28 items were presented on a 5-point Likert scale ranging from 1 (*doesn’t describe me at all*) to 5 (*describes me very well*), with higher values indicating greater empathy. In the current sample, Cronbach’s α was 0.78 for the total score.

To measure participants’ affective appraisal of their counseling skills, we applied the *Positive Affect Negative Affect Schedule* (PANAS) [24] in its German version [25]. The scale consists of 20 items which cover positive affect (PA; e.g., “interested”) and negative affect (NA; e.g., “irritated”). Answers are given on a 5-point Likert scale from 1 (not at all true) to 5 (extremely). Higher values reflect a higher intensity of the related affective state. Following Lent et al. [10] and Hahn et al. [4], the instruction was adapted to the participants’ role (e.g., “Indicate to what extent in general as a counselor, i.e., whilst performing counseling related activities, you feel…”). In our sample, Cronbach’s α was 0.73 for PA, and 0.70 for NA.

To measure student’s motivation to improve their own counseling skills, we adapted two scales of the *Patterns of Adaptive Learning Scales* (PALS) [26]: *Mastery Goal Orientation-Revised*, which assesses students’ determination to increase their perceived competence on four items (e.g., “One of my goals is to master a lot of new counseling skills this year.”), and *Performance Approach Goal Orientation-Revised*, which measures students’ determination to perform well in front of others with four items (e.g., “One of my goals is to show others that I’m good at my counseling class work.”). Answers are given on a five-point Likert scale, ranging from 1 (*not at all true*) to 5 (*very true*), with higher values indicating higher motivation to master counseling skills and perform well in front of others, respectively. In our sample, Cronbach’s α was 0.82 for the mastery scale and 0.71 for the performance scale.

### Data analysis

R (Version 4.1.3) [27] was used for data analysis. The level of significance was set at *α* = 0.05.

#### Missing data

Missing values amounted to 1,8%. Little’s [28] Missing Completely at Random (MCAR) test indicated that the data were missing completely at random (χ²(2045) = 2026, *p* =.613), for which reason missing values were replaced via imputation of individual means. For those cases in which values of entire subscales were missing, mean imputation was not possible. If this applied to participants in more than two subscales, they were excluded from further analyses (*n* = 5, post-training).

#### Cut-off criteria and fit measures

We applied R’s “lavaan” package [29] for.

confirmatory factor analysis (CFA) to analyze the fit of a three-factor model based on the basic CASES-R subscales by Hahn and colleagues [4]. To test our data for multivariate normality, Mardia’s multivariate kurtosis test [30], Mardia’s multivariate skewness test [30], Henze-Zirkler’s consistent test [31], and Doornik-Hansen omnibus test [32] were conducted in R with the help of the “MVN” package [33]. As all four tests were statistically significant (*p* <.001), the assumption of multivariate normality was rejected, and maximum likelihood estimations were conducted with robust standard errors and a Sartorra-Bentler scaled test statistic [34]. To evaluate the model fit, we used the robust χ2-test and the ratio of χ2/df, the comparative fit index (CFI), the Tucker-Lewis Index (TLI), the standardized root mean square residual (SRMR), and the root mean square error of approximation (RMSEA). Fit measures were evaluated in accordance with generally accepted standards [35–37] and in line with the criteria applied in the previous validation of German CASES-R by Hahn and colleagues [4]. Thus, a ratio of χ2/df below 3 indicates acceptable fit and a ratio below 2 a good fit. CFI and TLI values above 0.90 indicate an acceptable fit and values above 0.95 indicate a good fit. For the RMSEA, values below 0.05 indicate a good fit and values below 0.08 indicate an acceptable fit. SRMR values should be below 0.05 for a good fit, while values below 0.10 are acceptable.

#### Reliability

Internal consistencies were evaluated by computing the subscales’ Cronbach’s *α*. According to Cronbach [38], high values of *α* are considered desirable, and values above 0.70 are frequently reported to be acceptable [39]. However, as high Cronbach’s *α* values do not necessarily imply unidimensionality of the underlying construct but reflect that each item has shared variance with other items of a scale, we follow Taber’s [40] recommendation and report Cronbach’s *α* for each subscale rather than for the three CASES-R scales combined.

#### Validity

Pearson’s correlations were calculated between the three CASES-R scales and related constructs to evaluate convergent validity (i.e., GSE) and criterion validity (i.e., empathic concern, positive and negative affectivity, mastery orientation, performance orientation).

#### Further analyses

Through analysis of variance (ANOVA), we investigated the effects of students’ field of study (i.e., psychology or education) and time of assessment (i.e., before or after counseling training) on the reported CSE. This served to evaluate the instrument’s sensitivity to change.

## Results

### Confirmatory factor analysis

The CFA revealed six items loading on the factor EIS-R, six items loading on the factor SM-R, and three items loading on the factor AS-R (see Table 1). The overall fit of the model was acceptable to good for most of the indicators. While the model test was significant (*χ²*(87) = 168.46, *p* <.001), the *χ²*/df ratio remained below 2, nevertheless indicating a good fit. The robust CFI indicated an acceptable level of fit (CFI = 0.908), while the robust TLI and the robust RMSEA approached an acceptable fit (TLI = 0.890; RMSEA = 0.086, 90% CI [0.066, 0.105]). The SRMR indicated an acceptable fit (SRMR = 0.064). Overall, the three-factor solution showed an acceptable model fit. To ensure that each has sufficient variability to precisely estimate the latent variables, that is CSE in *exploration and insight skills*, *action skills*, and *session management*, we examined factor loadings and item variances (see Table 1). Sufficient factor loadings confirmed that our items were meaningful indicators for EIS-R, AS-R, and SM-R, contributing to the measurement of CSE. We found moderate levels of variability in participants’ responses on each item (see item variance in Table 1), indicating the scales’ ability to discriminate between participants with different levels of CSE. There were significant covariances between all latent variables, with standardized correlation coefficients ranging between 0.68 and 0.89 (all *p*’s < 0.001). EIS-R was positively correlated with AS-R (*r* =.74) and SM-R (*r =*.68), and AS-R with SM-R (*r* =.89).

**Table 1:** Item factor loadings and item variances

Short description of item	EIS-R	AS-R	SM-R	Var
1. Attending	0.354			0.875
2. Listening	0.593			0.649
3. Restatements	0.498			0.752
4. Reflection of feelings	0.696			0.516
5. Challenges	0.766			0.413
6. Interpretations	0.835			0.304
7. Direct guidance		0.745		0.444
8. Role-play and behavior rehearsal		0.713		0.492
9. Homework		0.704		0.504
10. Keeping sessions on track			0.556	0.690
11. Responding with helping skill			0.776	0.398
12. Knowing what to do or say next			0.759	0.423
13. Setting realistic goals			0.846	0.285
14. Conceptualization of client			0.729	0.469
15. Awareness of own intentions			0.710	0.497

*Notes*. EIS-R = Exploration and Insight Skills-Revised; AS-R = Action Skills-Revised; SM-R = Session Management-Revised; Var = item variance. All factor loadings and all item variances are standardized and significant at *p* <.001

### Reliability

The internal consistencies (Cronbach’s *α*) in this sample were good or acceptable for all factors (Total *α* = 0.81, EIS-R; *α* = 0.80, AS-R; *α* = 0.77, SM-R; *α* = 0.87).

### Convergent validity

There was a small but significant correlation between overall CSE and GSE (*r* =.23, *p* <.01).

### Criterion validity

Table 2 shows the correlations between CSE and the related constructs. As expected, we found a small yet significant correlation between CSE and with positive affect during counseling activities. No significant relationship was found between overall CSE and empathy, the motivation to master counseling skills, the motivation to perform well in counseling tasks, and negative affect during counseling activities. However, on the level of individual CASES-R scales, *exploration and insight skills* self-efficacy and *action skills* self-efficacy were significantly correlated with empathy, and *action skills* self-efficacy with the motivation to perform well. Furthermore, higher scores in *session management* self-efficacy were significantly associated with less negative affectivity towards counseling tasks (see Table 2).

**Table 2:** Correlations between CASES-R scales and validation measures (*n* = 163)

	CASES-R	EIS-R	AS-R	SM-R
GSE	0.23**	0.24**	0.16*	0.19*
IRI	0.14	0.21**	0.16*	0.04
MGO-R¹	0.07	0.14	0.05	0.09
PGO-R	0.11	0.02	0.16*	0.13
PANAS-NA	− 0.14	− 0.11	− 0.03	− 0.18*
PANAS-PA	0.22**	0.22**	0.19*	0.16*

*Notes*. CASES-R = CASES-R total mean, EIS-R = Exploration and Insight Skills-Revised, AS-R = Action Skills-Revised, SM-R = Session Management-Revised, GSE = general self-efficacy, IR = Interpersonal Reactivity Index, MGO-R = Mastery Goal Orientation-revised, PGO-R = Performance Approach Goal Orientation-revised, PANAS-NA = Negative Affect, PANAS-PA = Positive Affect. ¹ *n* = 162. * *p* <.05, ** *p* <.01, *** *p* <.001

### Increase with training and further analyses

**Fig. 2:**
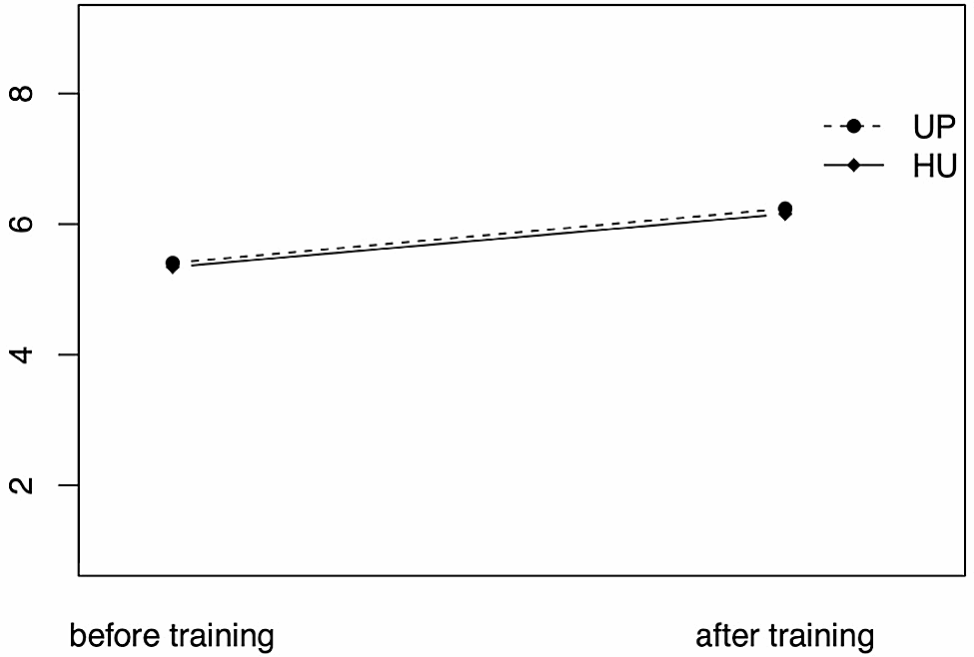
ANOVA of mean CSE by measurement time and university. UP = University of Potsdam, psychology students; HU = Humboldt University of Berlin, education students. CSE mean scores on a scale from 0 to 9

The ANOVA results were overall significant (*F*(3, 309) = 14.14, *p* <.001, *R²*_adj_ = 0.112). There was no significant main effect of field of study (*F*(1, 309) = -0.34, *p* =.73), indicating that students enrolled in psychology did not report higher CSE (*M* = 5.79, *SD* = 1.27) than students enrolled in education (*M* = 5.74, *SD* = 1.14). There was a significant main effect of time (*F*(1, 309) = 42.27, *p* <.001), indicating that students reported higher CSE after training (*M* = 6.18, *SD* = 1.05) than before (*M* = 5.37, *SD* = 1.16). There was no interaction between field of study and measurement time (*F*(1, 309) = 0.01, *p* =.942), indicating that students of both fields profited similarly from the counseling training they received (see Fig. 2). There was no significant difference in general self-efficacy between students enrolled in psychology and education (*t*(311) = 0.29, *p* =.78).

## Discussion

The current study aimed to validate the three CASES-R scales on trainees’ perceived ability in basic counseling skills. We confirmed the three-factor solution of the revised CASES-R scales [4], found good internal consistency, and yielded first information on validity and sensitivity to change.

### Factor structure and model fit

Aiming to evaluate a clear and economical measurement structure based on prior findings concerning the factor structure of CSE, we decided to analyze model fit using CFA. Item-level variances and factor loadings provided clear support for the latent variables EIS-R, AS-R, and SM-R. However, in contrast with structural equation modeling (SEM), items are only permitted to load on one factor in CFA, a severe restriction which can result in lower model fit when using this more conservative approach [41, 42]. Hahn and colleagues [4], for example, reported an improved model fit when investigating the factor structure of the CASES-R scales by instead of CFA applying a bifactor-ESEM model to their data. This may explain why the CFI fit index of our model was only acceptable, with RMSEA and TLI values that closely approximated the acceptable range. Not allowing cross-loadings can also contribute to an overestimation of factor correlations [41, 42], which is likely to explain the relatively high and significant standardized correlations found between EIS-R, AS-R, and SM-R in our study. Nonetheless, despite using the more rigorous statistical procedure, we were overall able to confirm the factor structure found by Hahn et al. [4], suggesting that exploration and insight skills, action skills, and session management skills are distinct components of basic counseling competencies. A SEM approach may have resulted in improved model fit, however, a larger sample size than ours would have been necessary. Given our focus on investigating CSE in early stages of training, we did not evaluate the complete CASES-R but only applied the scales regarding self-efficacy in basic counseling skills. While other researchers have similarly applied only specific CASES scales [e.g., 15], a formal validation of this use of the instrument has not previously been conducted.

### Reliability

Good internal consistency, measured with Cronbach’s *α*, suggests that the CASES-R scales are reliable. However, it has been criticized that reporting cut-off values for Cronbach’s *α* is not enough to prove reliability, highlighting the necessity of interpreting and discussing the obtained values in context. In our study, it is unlikely that internal consistency is artificially elevated by redundant items, as we applied the revised scales of CASES-R that were previously rigorously shortened by Hahn and colleagues [4]. Neither is the good internal consistency of our scales a product of assessing an overly narrow construct, as CSE of basic skills encompasses a variety of features that are distinct from one another. It is therefore reasonable to propose that the internal consistency of our three CASES-R scales reflects satisfactory reliability. As this is not only the case for the overall score but for each of the three scales individually, we hereby confirmed Lent’s suggestion that the three parts of the instrument may be applied individually [10, 15].

### Validity

In contrast to the moderate correlation found by Hahn and colleagues [4], we only found a small correlation between CSE and general self-efficacy. First, it should be noted that the sample in Hahn’s study was much larger than in ours. Furthermore, their study applied all five scales of the CASES-R, assessing CSE more globally than our measurement of only basic counseling skills with three subscales of the CASES-R. Grether and colleagues [43] suggest that while GSE and domain-specific self-efficacies are thought to impact each other and be related, they are not the same construct. In a broad investigation on GSE, Luszcynska and colleagues [6] found low correlations between GSE and specific behaviors and skills, suggesting that GSE may be closer to other global constructs. This may explain why our specialized measure of CSE was not highly related to GSE, indicating its lower suitability to confirm convergent validity in our study. For this purpose, we recommend the use of existing measures of CSE, such as the COSE [44], or occupational self-efficacy for this purpose [e.g., 10] in future research. It has been suggested that occupational self-efficacy can be substituted with academic self-efficacy in a student sample [43].

We found a small, significant correlation between higher CSE and positive affectivity towards counseling tasks. Though in line with prior research, the relationship was smaller than the moderate correlations we expected and may have been in part due to our smaller sample size and the early beginner stage of our participants. Unlike previous findings, in which higher CSE was related to lower negative affectivity and vice versa [4, 10], in our sample the relationship was less pronounced: We only found this association in *session management* self-efficacy, indicating that students reporting higher CSE managing counseling sessions, i.e., keeping track of time, were experiencing lower negative affect in counseling activities. Besides potentially being modest in their reported CSE, students may not experience strong negative affect in their deficient counseling skills, as they might simply be accepting the need for improvement as a part of the learning process in a university setting.

We did not find a significant correlation between CSE in basic skills and empathetic concern, which was unexpected in the light of previous findings [9, 45]. However, on the level of individual scales, it was the more technical scale for *session management* self-efficacy that was not correlated with empathy, whereas self-efficacy in exploring clients’ problems, generating insights, as well as developing actions and goals were significantly related to empathy.

Furthermore, we did not find the expected relationship between overall CSE and the motivation to master counseling skills, as well as the motivation to perform well before others in counseling tasks. Given the typically performance-oriented university class setting, we expected CSE to be related to stronger goal motivation, however, only found this effect in the scale for self-efficacy in *action skills*. It is possible that the fact that the university classes were not graded resulted in a learning environment with low performance pressure. Concerning goal orientation, Lent [10] found significant relations between CSE and interest in counseling activities as well as counseling career goals. Our results may furthermore be due to the heterogeneity of career orientations in our sample or the training stage of our participants, many of whom were in very early stages of their education. Overall, the small but significant correlations we found between the CASES-R scales and related constructs highlight the need for further studies to evaluate the validity of the instrument.

### Levels of experience and increase with training

There was heterogeneity in our sample since participating students were enrolled in different programs, namely psychology, special education, and rehabilitation education. Psychology students were furthermore enrolled in master’s degree programs while education students were enrolled to obtain their bachelor’s degree. It is possible that the heterogeneity of our sample may have negatively impacted our findings, for example resulting in weaker correlations with related constructs than if a homogeneous sample in terms of education and skill had been used. Yet, we confirmed that there was neither a significant difference in CSE nor in general self-efficacy between students of different fields and levels of studying. It is nevertheless likely that students in our sample had different levels of skills regarding counseling activities, either due to university courses or internships in therapy or counseling settings. As we did not objectively assess competence, e.g., by independent raters, it is not possible to relate differences in self-reported CSE to differences in students’ skill levels. However, our analyses revealed that psychology and education students profited equally from the training of specific counseling skills, in terms of their counseling self-efficacy. This evidence suggests that the CASES-R scales are suited to precisely assess CSE at beginner level in different career tracks, and that they are sensitive to changes in confidence and experience.

### Fields of application

A range of potential applications for the CASES-R measuring basic skills is conceivable. Like in our study, the instrument can inform the development of a training curriculum for prospective counselors, as it encompasses an overview of core counseling skills. It could be applied to evaluate the effectiveness of counseling trainings regarding the outcome of CSE. Furthermore, being a particularly time-efficient tool, it may be applied regularly to monitor the development of prospective counselors over the course of skills trainings. Here, parallel measurements of other constructs, such as motivational factors or career orientation, could particularly enhance our current understanding of how early counseling trainees develop skills and professional confidence. The CASES-R may also be interesting for clinical practice. With its economic application, the instrument could be a valuable tool for counselors’ self-reflection, yet also be applied in contexts such as supervision, where it could help supervisors identify specific areas for development.

### Limitations

While it is a strength of our study that we investigated beginner-level CSE more broadly across two different universities and three different fields of study, this is also a limitation. Counseling trainings were conducted by six different lecturers with potentially varying teaching methods, styles, or practical exercises. Furthermore, it is important to note that all measures were assessed by self-report. We assessed students’ perceived self-efficacy and did not apply an objective measure to assess competence. While confidence in one’s own counseling skills has previously been related to competence [46], this should be addressed specifically in future research, focusing on the early training phase. Further limitations include our moderate sample size, as well as the lack of diversity that is typically found in non-randomized samples such as our group of mostly female, young adult participants. Although the field of mental health care in Germany is reflective of these characteristics [47], it nevertheless limits the generalizability of our findings.

## Conclusions

This study contributes to the understanding of the development of relevant competencies in prospective counselors by providing a precise and economically applicable instrument to measure CSE at early training stages. We found support for the factor structure and reliability of the German version of the three CASES-R scales that assess self-efficacy in basic counseling settings. Relevant areas of application for the instrument include the evaluation of the effectiveness of counseling skills trainings, such as university courses, as well as the monitoring of students’ self-efficacy for research purposes, to better understand this important aspect of counselors’ professional development. Future research should further evaluate the validity of the instrument by investigating its relationship with existing measures of CSE, as well as with objective ratings of counseling competence.

### Electronic supplementary material

Below is the link to the electronic supplementary material.

Supplementary Material 1

## Data Availability

The datasets generated and/or analyzed during the current study are not publicly available due to ethical restrictions but are available from the corresponding author on reasonable request. The German version of the CASES-R scales will also be made available on request from the corresponding author.

## Abbreviations

CASES/ CASES-R: Counselor Activity Self-Efficacy Scales/ -revised
CSE: Counselor self-efficacy
HS: *Helping Skills* self-efficacy
SM: *Session Management* self-efficacy
CC: *Counseling Challenges* self-efficacy
IS: Insight Skills (CASES subscale)
ES: Exploration Skills (CASES subscale)
EIS-R: Exploration and Insight Skills (CASES-R subscale)
AS/AS-R: Action Skills (CASES subscale)/ -revised (CASES-R subscale)
SM/SM-R: Session Management (CASES subscale)/ -revised (CASES-R subscale)
RC/RC-R: Relationship Conflict (CASES subscale)/ -revised (CASES-R subscale)
CD/CD-R: Client Distress (CASES subscale)/ -revised (CASES-R subscale)
EFA: Exploratory factor analysis
ESEM: Exploratory structure equation modeling
CFA: Confirmatory factor analysis
UP: University of Potsdam
HU: Humboldt Universität zu Berlin
GSE: General Self-Efficacy
IRI: Interpersonal Reactivity Index
PANAS– PA/NA: Positive Affect Negative Affect Schedule– Positive Affect/ Negative Affect
PALS: Patterns of Adaptive Learning Scales
MGO-R: Mastery Goal Orientation-revised
PGO-R: Performance Approach Goal Orientation-revised
CFI: Comparative fit index
TLI: Tucker-Lewis Index
SRMR: Standardized root mean square residual
RMSEA: Root mean square error of approximation
ANOVA: Analysis of variance
