# Supplementary material for: Basic counseling skills in psychology and teaching: validation of a short version of the counselor activity self-efficacy scales
Source: BMC Psychol. 2024 Jan 18;12:32. doi: 10.1186/s40359-023-01506-7 (PMC10797791; doi:10.1186/s40359-023-01506-7)
Supplement: Supplementary file 1 — Supplementary Material 1 [file 40359_2023_1506_MOESM1_ESM.docx]

***Supplementary Material 1***

*German CASES-R for basic counseling skills*

| **Name** | **CASES-R subscale** | **Original item number^1^** |
| --- | --- | --- |
| Attending | EIS-R | 2.5^a^ |
| Listening | EIS-R | 2.2^a^ |
| Restatements | EIS-R | 2.4^a^ |
| Reflection of feelings | EIS-R | 2.3^a^ |
| Challenges | EIS-R | 1.4^a^ |
| Interpretations | EIS-R | 1.2^a^ |
| Direct guidance | AS-R | 3.3^a^ |
| Role-play and behavior rehearsal | AS-R | 3.2^a^ |
| Homework | AS-R | 3.4^a^ |
| Keep sessions on track | SM-R | 8^b^ |
| Respond with helping skill | SM-R | 7^b^ |
| Next step | SM-R | 2^b^ |
| Setting realistic goals | SM-R | 6^b^ |
| Conceptualization of client | SM-R | 4^b^ |
| Own intentions | SM-R | 9^b^ |

*Notes.* Overview over the items of the short version of the CASES-R for basic skills. The name of each item is provided, the respective subscale, and which item in the original CASES instrument by Lent et al. (2003) it corresponds to.

^1^ Number of the item as it appears in the original CASES (Lent et al., 2003)

^a^ see *table 1* in Lent et al. (2003) for a full description of the item

^b^ see *table 2* in Lent et al. (2003) for a full description of the item

*The German version of the CASES-R is available to download for research purposes here:* <https://www.uni-potsdam.de/de/clinical-psychology-psychotherapy/forschungsschwerpunkte/i-psychotherapeutische-kompetenzen-1/psychotherapeutische-kompetenzmasse>
